# Comparison of Proton Pump Inhibitor and Histamine-2 Receptor Antagonist in the Prevention of Recurrent Peptic Ulcers/Erosions in Long-Term Low-Dose Aspirin Users: A Retrospective Cohort Study

**DOI:** 10.1155/2014/693567

**Published:** 2014-09-11

**Authors:** Wen-Chi Chen, Yun-Da Li, Po-Hung Chiang, Feng-Woei Tsay, Hoi-Hung Chan, Wei-Lun Tsai, Tzung-Jiun Tsai, E-Ming Wang, Jin-Shiung Cheng, Kwok-Hung Lai

**Affiliations:** ^1^Division of Gastroenterology, Department of Medicine, Kaohsiung Veterans General Hospital, No. 386, Ta-Chung 1st Road, Kaohsiung 813, Taiwan; ^2^School of Medicine, National Yang-Ming University, No. 155, Section 2, Linong Street, Taipei 112, Taiwan; ^3^Department of Chemistry, College of Science, National Kaohsiung Normal University, No. 62, Shenjhong Road, Kaohsiung 824, Taiwan; ^4^Department of Sport, Health & Leisure, Cheng Shiu University, No. 840, Chengcing Road, Kaohsiung 833, Taiwan

## Abstract

*Background*. Proton pump inhibitor and histamine-2 receptor antagonist can prevent aspirin-related ulcers/erosions but few studies compare the efficacy of these two agents. *Aims*. We evaluated the efficacy of omeprazole and famotidine in preventing recurrent ulcers/erosions in low-dose aspirin users. *Methods*. The 24-week clinical outcomes of the patients using low-dose aspirin for cardiovascular protection with a history of ulcers/erosions and cotherapy of omeprazole or famotidine were retrospectively reviewed. The incidence of gastrointestinal symptoms, recurrent ulcers/erosions, erosive esophagitis, gastrointestinal bleeding, and thromboembolic events was analyzed. *Results*. A total of 104 patients (famotidine group, 49 patients; omeprazole group, 55 patients) were evaluated. Famotidine group had more gastrointestinal symptoms episodes than omeprazole group (46.9% versus 23.6%, *P* = 0.01). Fifteen famotidine group patients and 5 omeprazole group patients had recurrent ulcers/erosions (30.6% versus 9.1%, *P* = 0.005). Lanza scale was significantly lower in omeprazole group than in famotidine group (1.2 ± 0.7 versus 1.7 ± 1.1, *P* = 0.008). Only 1 famotidine group patient had ulcer bleeding. The incidences of erosive esophagitis and thromboembolic events were comparable between both groups. *Conclusions*. Omeprazole was superior to famotidine with less gastrointestinal symptoms and recurrent ulcers/erosions in patients using 24-week low-dose aspirin. The risk of erosive esophagitis, gastrointestinal bleeding, and thromboembolic events was similar between both groups.

## 1. Introduction

Aspirin is a nonsteroidal anti-inflammatory drug (NSAID) with antiplatelet effect and has been widely used in primary or secondary prevention of cardiovascular and cerebrovascular events at a low dose of 75 to 325 mg daily [1–3]. Low-dose aspirin reduces prostaglandin levels in stomach and duodenum [4] and induces gastric and duodenal mucosal injury, ulcer formation, or bleeding [5, 6]. Proton pump inhibitors (PPI) have gastroduodenal protective effect of aspirin-related ulcers [7]. To prevent mucosa damage caused by aspirin, ACC/AHA guideline suggests cotherapy of aspirin and PPI after unstable angina or non-ST elevation myocardial infarction in patients with previous gastrointestinal bleeding (GIB) [8]. Recently, a nationwide cohort study in Taiwan also found that aspirin plus PPI was superior to clopidogrel alone or clopidogrel plus PPI in terms of reducing the risk of GIB while maintaining the cardiovascular protective effect [9].

However, long-term PPI use may be associated with some adverse effects. Increased risk of osteoporosis and fracture is observed in patients using long-term PPIs [10]. Besides, patients using long-term PPIs are at a risk of hypergastrinemia, hypochlorhydria, hypomagnesaemia, malabsorption, and infectious diarrhea [11]. The safety of long-term PPIs should be concerned in aspirin users in addition to the gastroduodenal protective effect of PPIs.

*Helicobacter pylori* (*H. pylori*) infection increases the risk of peptic ulcer diseases in patients using NSAIDs including aspirin [12].* H*.* pylori* eradication can prevent gastropathy and should be undertaken in patients with a history of peptic ulcers and who need long-term aspirin therapy [13]. After eradication of* H*.* pylori*, cotherapy with a PPI is still suggested in high-risk aspirin users [13]. However, whether use of other acid suppressants such as a histamine-2 receptor antagonist (H2RA) is feasible in this case is seldom investigated.

H2RAs had also been proved to reduce the gastric toxicity of aspirin [14]. Although PPIs have a stronger acid suppression capacity than H2RAs, patients using H2RAs are at a lower risk of pneumonia and* Clostridium difficile* infection than PPIs users [15]. The efficacy of PPIs and H2RAs in the prevention of recurrent ulcers/erosions in aspirin users is rarely compared. Omeprazole and famotidine are both potent acid suppressants which decrease the incidence of recurrent ulcers/erosions in patients taking long-term aspirin [14, 16, 17]. This study aimed at comparing the protective effect of omeprazole and famotidine in long-term low-dose aspirin users with a history of aspirin-related peptic ulcers. All the patients with* H*.* pylori* infection in this cohort received eradication therapy of* H*.* pylori* as suggested by the guidelines.

## 2. Materials and Methods

### 2.1. Patients

This retrospective cohort study was conducted at Kaohsiung Veterans General Hospital and was approved by the Institutional Review Board (VGHKS13-CT12-07). We reviewed the medical records of the patients using long-term low-dose aspirin from January 2008 to December 2012. Patients were considered to be enrolled into this study if they met the following inclusion criteria: (1) age equal to or more than 20 years, (2) use of aspirin 75 to 325 mg daily for primary or secondary prevention of coronary artery disease or cerebral vascular accident, (3) a history of aspirin-related peptic ulcers/erosions, (4) no gastroduodenal ulcers/erosions or erosive esophagitis on esophagogastroduodenoscopy (EGD) at the initial analysis point, (5) concomitant use of famotidine 40 mg daily or omeprazole 20 mg daily for the prevention of recurrent aspirin-related ulcers, (6) undergoing EGD at around 24 weeks from the initial analysis point, and (7) being negative for* H. pylori* infection by a histology, rapid urease test, or urea breathing test at the initial analysis point. The exclusion criteria were (1) active malignancy; (2) a history of surgery for esophagus, stomach, or duodenum; (3) concomitant use of anticoagulants, thienopyridines, misoprostol, antacid, and mucosa protecting agents; (4) use of NSAIDs ≥1 week; (5) use of aspirin, famotidine, or omeprazole for less than 6 months from the initial analysis point; (6) pregnancy; and (7) chronic renal insufficiency.

### 2.2. Methods

According to the standard treatment in our institute, patients with a peptic ulcer/erosion history who underwent long-term aspirin therapy would receive concomitant PPI or H2RA therapy. Additionally, follow-up endoscopy was performed 6 months later and whenever severe dyspepsia or GIB occurred and* H. pylori* testing was also conducted. In this study, we retrospectively reviewed the demographic data of the patients including age, gender, personal habits (cigarette, alcohol, coffee, and tea consumption), concomitant diseases, indications of aspirin use, doses of aspirin, concomitant medications including steroids and short-term (<1 week) NSAIDs during the study period, history of upper GIB if present, history of* H*.* pylori* infection, and the eradication therapies of* H. pylori*, and the findings of follow-up EGD were retrieved from the medical records. Besides, the incidences of gastrointestinal symptoms including epigastralgia, bloating, nausea and vomiting, recurrent peptic ulcers/erosions, erosive esophagitis, peptic ulcer bleeding, cerebral vascular accident, transient ischemic attack, and acute coronary syndrome were assessed.

### 2.3. Definition

An ulcer was defined as a mucosal break more than or equal to 3 mm of its longest diameter found on EGD and was estimated by biopsy forceps. An erosion was defined as a mucosal break less than 3 mm of its longest diameter. The severity of gastroduodenal mucosal injury was estimated using Lanza scale (grade 0: no visible lesions; grade 1: mucosal hemorrhage only (≤25); grade 2: 1-2 erosions, or >25 hemorrhages; grade 3: 3–9 erosions; grade 4: ≥10 erosions or an ulcer) [18]. Consumption of coffee, tea, and alcohol was defined as consumption of coffee, tea, or alcohol more than or equal to 4 days per week. Cigarette consumption was defined as daily smoking during the study period.* H*.* pylori* infection status was determined by rapid urease test, urea breath test, or histology results at the initial analysis point. Upper GIB was defined as patients presented with hematemesis, tarry stool, or hematochezia or bleeding found on endoscopic examination. Acute coronary syndrome was defined as occurrence of unstable angina or acute myocardial infarction. Cerebral vascular accident was defined as patients presenting with typical neurological symptoms such as hemiplegia, dysphagia, and slurred speech or typical image findings. Transient ischemic attack was defined as patients presented with typical neurological symptoms and fully recovered within 24 hours.

### 2.4. Study End Points

The primary end point of this study was recurrent ulcers or erosions found on endoscopic examination. The secondary end points were occurrence of gastrointestinal symptoms, erosive esophagitis, upper GIB, and thromboembolic events including acute coronary syndrome, ischemic stroke, or transient ischemic attack.

### 2.5. Statistical Analysis

Demographic data and the occurrence of primary and secondary end points were compared between both groups. Categorical data were compared using Chi-square or Fisher's exact tests when appropriate. Continuous variables with normal distributions were compared using independent Student's *t*-test. Continuous variables without normal distributions were compared using Mann-Whitney *U* test. Univariate logistic regression analysis was performed to examine the variables significantly associated with recurrent peptic ulcers or erosions. Significance was defined as *P* < 0.05 for all two-tailed tests. All analyses were conducted by using SPSS software (version 12; SPSS Inc., Chicago, IL).

## 3. Results

### 3.1. Characteristics of Patients

Between January 2008 and December 2012, a total of 104 eligible patients (famotidine group, 49 patients; omeprazole group, 55 patients) using long-term low-dose aspirin were analyzed. All the patients received routine* H*.* pylori* testing because they had a history of aspirin-related peptic ulcers/erosions. Of the 19 famotidine group patients with a history of* H. pylori* infection,* H*.* pylori* was successfully eradicated by using 7-day triple therapy (PPIs, amoxicillin, and clarithromycin for 7 days) (11 patients) and 14-day hybrid therapy (PPIs and amoxicillin for 14 days plus clarithromycin and metronidazole for 7 days) (8 patients). Of the 14 omeprazole group patients with a history of* H*.* pylori* infection,* H*.* pylori* was successfully eradicated by using 7-day triple therapy (5 patients) and 14-day hybrid therapy (9 patients). All the patients were negative for* H*.* pylori* infection by a histology, rapid urease test, or urea breathing test at the initial analysis point. All the patients with a history of* H*.* pylori* infection were still negative for* H*.* pylori* testing in the endpoint of analysis. The demographic data of famotidine group and omeprazole group showed similar age, gender, duration from the initial analysis point to follow-up endoscopy, and consumption of smoking, alcohol, coffee, and tea. The proportions of history of upper GIB, history of* H*.* pylori* infection, indications of aspirin therapy, and concomitant use of short-term NSAIDs and steroids were also comparable between both groups (Table 1).

### 3.2. Incidence of Gastrointestinal Symptoms and Bleeding

Twenty-three episodes of gastrointestinal symptoms were observed in famotidine group: dyspepsia (8 patients), acid reflux (8 patients), epigastralgia (6 patients), and belching (1 patient). One patient in famotidine group presented with gastric ulcer bleeding (Table 2). Thirteen episodes gastrointestinal symptoms were found in omeprazole group patients: dyspepsia (5 patients), acid reflux (2 patients), epigastralgia (5 patients), and belching (1 patient). No patient in omeprazole group had GIB. Significantly more episodes of gastrointestinal symptoms were found in famotidine group patients than in omeprazole group patients (46.9% versus 23.6%, *P* = 0.01).

### 3.3. Findings of Follow-Up Endoscopy

Patients in famotidine group had a significantly higher incidence of recurrent ulcers/erosions than patients in omeprazole group (30.6% versus 9.1%, *P* = 0.005). Of the patients with recurrent ulcers/erosions, asymptomatic ulcers/erosions were found in 9 of 15 (60.0%) famotidine group patients and 4 of 5 (80.0%) omeprazole patients. Five of 19 patients (26.3%) with a history of* H*.* pylori* infection in famotidine group had recurrent ulcers/erosions and 1 of 14 patients (7.1%) with a history of* H*.* pylori* infection in omeprazole group had recurrent ulcers/erosions (*P* = 0.2). In ulcer analysis, 10 patients in famotidine group (20.4%) and 4 patients in omeprazole group (5.5%) had recurrent ulcers (*P* = 0.04). We evaluated the severity of gastroduodenal mucosal injury using Lanza score and found that omeprazole also showed a better protective effect than famotidine (1.2 ± 0.7 versus 1.7 ± 1.1, *P* = 0.008). Besides, erosive esophagitis was present in 7 patients (14.3%) in famotidine group and 7 patients (12.7%) in omeprazole group (*P* = 1.0) (Table 3).

Univariate logistic regression analysis of variables including age ≥60 years, gender, indications of aspirin use, history of upper GIB, history of* H*.* pylori* infection, use of NSAIDs <1 week, and steroids therapy found that omeprazole therapy was the only factor associated with a lower risk of aspirin-related ulcers/erosions (relative risk: 0.2; 95% confidence interval: 0.08–0.7; *P* = 0.008) (Table 4).

### 3.4. Incidence of Thromboembolism

In omeprazole group, 4 patients had acute coronary syndrome (unstable angina, 2 patients; acute myocardial infarction, 2 patients) while no patient in famotidine group had thromboembolic event. The incidence of thromboembolic events was comparable between both groups (Table 5).

## 4. Discussion

The current study revealed that omeprazole was superior to famotidine in the prevention of recurrent ulcers/erosions in long-term low-dose aspirin users free for active* H*.* pylori* infection. A very low upper GIB rate was observed in this cohort. Famotidine group patients had more episodes of gastrointestinal symptoms than omeprazole group patients. Besides, the incidences of erosive esophagitis and thromboembolic events were similar between omeprazole and famotidine group patients.

Few studies compared the efficacy of PPIs and H2RAs in the prevention of recurrent peptic ulcers due to long-term aspirin use. Ng et al. found that high-dose famotidine (80 mg daily) was inferior to pantoprazole (20 mg daily) with a significantly higher incidence of aspirin-related ulcers/erosions or GIB [19]. However, only patients with dyspepsia, severe epigastric pain, or GIB underwent endoscopy and the incidence of asymptomatic ulcers/erosions might be underestimated. Tamura et al. evaluated patients taking low-dose aspirin with either standard-dose famotidine (40 mg daily) or lansoprazole (15 mg daily). Significantly, more patients in famotidine group (48.4%) presented with gastroduodenal erosions than patients in lansoprazole group (17.0%) and no ulcer was found in both groups [20]. Nevertheless, the treatment duration of acid suppressants was not identical between both groups. Besides, part of the patients took antiplatelet agents other than aspirin or anticoagulants and nearly half of the patients were positive for urinary* H. pylori* antibody while only about 10% of the patients had received eradication therapy of* H*.* pylori*. The incidence of aspirin-related ulcers might be overestimated because of high* H*.* pylori* infection rate and concomitant use of antiplatelet agents or anticoagulants in some patients. Another study by Ng et al. found that esomeprazole was superior to famotidine in preventing upper GIB, perforation, or obstruction from ulcers/erosions in patients with acute coronary syndrome or myocardial infarction [21]. Unfortunately, a combination of aspirin, clopidogrel, and enoxaparin or thrombolytics was used and the role of aspirin was not specifically investigated.

This study compared the protective efficacy of standard-dose famotidine with omeprazole after long-term low-dose aspirin use with the superiority that all patients were free for active* H. pylori* infection and all patients underwent follow-up endoscopy at the end of the study, which allowed us to estimate the exact incidence of aspirin-related erosions/ulcers. Patients in omeprazole group (9.1%) had a significantly lower incidence of ulcers/erosions compared with patients in famotidine group (30.6%), which was consistent with previous studies. The severity of gastroduodenal injury determined by using Lanza score was also less severe in omeprazole group than in famotidine group. During subgroup analysis, we found that the incidence of gastroduodenal ulcers in omeprazole group (5.5%) was significantly lower than that of famotidine group (20.4%). Our findings supported the ACC/AHA guideline that cotherapy of aspirin and PPI is the treatment of choice in patients with a history of peptic ulcer and who need long-term aspirin therapy.

Gastrointestinal mucosal injury associated with low-dose aspirin is often asymptomatic [22]. Although famotidine group patients had more frequent gastrointestinal symptoms than omeprazole group patients, 65% of the ulcers/erosions found on endoscopy were asymptomatic in this study. Our findings suggested that typical peptic ulcer symptoms such as dyspepsia and epigastralgia were not reliable predictors for recurrent gastroduodenal ulcers/erosions in low-dose aspirin users who were on concomitant acid suppressants. Actually, asymptomatic gastroduodenal erosions also can be associated with GIB [23]. Therefore, periodical endoscopy surveillance might be necessary in patients taking long-term low-dose aspirin even if concomitant acid suppressants are used.

The risk of GIB was very low and similar between patients using omeprazole (0%) and famotidine (2%) in our cohort. This result contradicted the study by Ng et al. in which 5 patients (7.7%) using famotidine had bleeding ulcer/erosion at 9, 2, 32, 16, and 36 weeks, respectively, while no patients using pantoprazole had ulcer/erosion bleeding [19]. It was possible that the study period of our study was 24 weeks and the incidence of GIB might increase after a longer observation period, especially in the patients using famotidine.

The risk of erosive esophagitis is not as high as that of peptic ulcers in aspirin users [24]. Nevertheless, erosive esophagitis is common in low-dose aspirin users with upper GIB [25]. In long-term aspirin user, erosive esophagitis was found in 4.4% of the patients taking famotidine compared with 19.0% of the patients taking placebo [14]. Rabeprazole can also reduce the risk of erosive esophagitis in aspirin user [26]. In this study, the incidence of erosive esophagitis was about 10% in both groups. However, we found that omeprazole group was associated with less incidence of acid reflux, the typical symptom of erosive esophagitis, than famotidine group although the case numbers were limited. A randomized control trial with a larger sample size is necessary to investigate the difference of incidence of erosive esophagitis between PPIs and H2RAs in long-term aspirin users.

For aspirin users, eradication of* H. pylori* can decrease the risk of recurrent ulcer bleeding to a very low extent [27]. Unfortunately,* H*.* pylori* eradication alone does not reduce the incidence of ulcers in patients already receiving long-term aspirin and continued PPI treatment is still necessary [13]. In our study, the incidence of recurrent erosions/ulcers was similar between omeprazole and famotidine group during subgroup analysis of the patients receiving eradication therapy of* H*.* pylori* but the power was limited. Further study is required to compare the efficacy of PPIs and H2RAs in the prevention of recurrent aspirin-related ulcers after eradication of* H*.* pylori*.

There were some limitations in our study. First, selection bias and missing data did exist in this retrospective cohort study and the final results might be biased. Second, although omeprazole patients had less gastrointestinal symptoms than famotidine group patients, the symptoms might be missed if they were not documented by the caring physicians. Third, the study period was only 24 weeks and a higher incidence of gastroduodenal ulcers/erosions would be expected after a longer observation period. Fourth, we could not exclude the possibility of false-negative* H. pylori* testing in omeprazole group patients even though all patients were negative for active* H*.* pylori* infection during the study period. Finally, although the risk of GIB and thromboembolic event was similar between omeprazole and famotidine group, the sample size of this cohort was not large enough and further studies are required to compare the incidence of GIB and thromboembolism between PPI and H2RA users.

## 5. Conclusions

In conclusion, this study suggested that cotherapy of omeprazole had less gastrointestinal symptoms and a better protective efficacy in the prevention of recurrent peptic ulcers/erosions than famotidine in patients using long-term low-dose aspirin. However, the risk of erosive esophagitis, GIB, and thromboembolic events was similar between these two therapeutic strategies in this cohort. Because of the high occurrence rate of asymptomatic ulcers/erosions, periodical endoscopic surveillance might be necessary in patients using cotherapy of acid suppressants and long-term low-dose aspirin.

## Figures and Tables

**Table 1:** Demographic data of patients taking long-term low-dose aspirin.

Variables	Famotidine group (*n* = 49)	Omeprazole group (*n* = 55)	*P* value
Age (year)	74.4 ± 10.5	73.3 ± 10.6	0.6
≥60 years old	44 (89.8%)	48 (87.3%)	0.7
Male gender	42 (85.7%)	42 (76.4%)	0.2
Time to follow-up EGD∗ (weeks)	25.0 ± 2.3	24.5 ± 2.1	0.2
Smoking	6 (12.2%)	8 (14.5%)	0.7
Alcohol consumption	2 (4.1%)	3 (5.5%)	1.0
Coffee consumption	4 (8.2%)	7 (12.7%)	0.4
Tea consumption	15 (28.6%)	8 (14.5%)	0.8
Cirrhosis	1 (2.0%)	1 (1.8%)	1.0
Hiatus hernia	18 (36.7%)	18 (32.7%)	0.7
History of upper GIB^†^	10 (20.4%)	12 (21.8%)	0.9
History of erosive esophagitis	8 (16.3%)	17 (30.1%)	0.08
History of *H*. *pylori* infection	19 (38.8%)	14 (25.5%)	0.1
Concomitant medication			
Steroids	3 (6.1%)	2 (3.6%)	0.6
Short-term NSAID^‡^	5 (10.2%)	7 (12.7%)	0.7

∗EGD: esophagogastroduodenoscopy.

^†^GIB: upper gastrointestinal bleeding.

^‡^NSAID: nonsteroidal anti-inflammatory drug.

**Table 2:** Sequelae of patients taking long-term low-dose aspirin.

	Famotidine group (*n* = 49)	Omeprazole group (*n* = 55)	*P* value
Gastrointestinal symptoms	23 (46.9%)	13 (23.6%)	0.01
Dyspepsia	8 (16.3%)	5 (9.1%)	0.3
Acid reflux	8 (16.3%)	2 (3.6%)	0.04
Epigastralgia	6 (12.2%)	5 (9.1%)	0.6
Belching	1 (2%)	1 (1.8%)	1.0
Peptic ulcer bleeding	1 (2%)	0 (0%)	0.5

**Table 3:** Follow-up endoscopic findings of patients taking long-term low-dose aspirin.

	Famotidine group (*n* = 49)	Omeprazole group (*n* = 55)	*P* value
Lanza scale	1.7 ± 1.1	1.2 ± 0.7	0.008
Gastroduodenal ulcer/erosion∗	15 (30.6%)	5 (9.1%)	0.005
Ulcer	10 (20.4%)	3 (5.5%)	0.04
Gastric ulcer^†^	6	2	
Duodenal ulcer	3	1	
Gastric ulcer & duodenal ulcer	1	0	
Erosion	5 (10.2%)	2 (3.6%)	0.2
Gastric erosion	5	1	
Gastric erosion & duodenal erosion	0	1	
Erosive esophagitis	7 (14.3%)	7 (12.7%)	1.0

∗Results were presented as the most severe mucosal injury found on endoscopy.

^†^One patient in famotidine group had gastric ulcer bleeding.

**Table 4:** Univariate analysis of risk factors for recurrent ulcers/erosions in long-term low-dose aspirin users.

Variables	Relative risk	95% confidence interval	*P* value
Age ≥ 60 years	0.4	0.1–1.6	0.2
Primary prevention	1.1	0.4–3.3	0.8
Male gender	2.5	0.5–11.6	0.3
Omeprazole group	0.2	0.08–0.7	0.008
Smoking	2.8	0.8–9.5	0.1
Alcohol consumption	3.0	0.5–19.3	0.2
Coffee consumption	0.4	0.05–3.2	0.4
Tea consumption	1.3	0.4–4.1	0.6
History of UGIB∗	0.6	0.2–2.3	0.5
History of *H*. *pylori* infection	0.9	0.3–2.6	0.9
Concomitant medication			
Steroids	1.1	0.1–10.0	1.0
Use of NSAID^†^ <1 week	1.5	0.4–6.0	0.6

∗UGIB: upper gastrointestinal bleeding.

^†^NSAID: nonsteroidal anti-inflammatory drug.

**Table 5:** Thromboembolic events in patients taking long-term low-dose aspirin.

	Famotidine group (*n* = 49)	Omeprazole group (*n* = 55)	*P* value
Total events	0	4 (7.3%)	0.1
Acute coronary syndrome	0	4	
Acute myocardial infarction	0	2	
Unstable angina	0	2	
Cerebral vascular accident	0	0	
Transient ischemic attack	0	0	

## Abbreviations

PPI: Proton pump inhibitor
H2RA: Histamine 2 receptor antagonist
GIB: Gastrointestinal bleeding
NSAID: Nonsteroidal anti-inflammatory drug
*H. pylori*: * Helicobacter pylori*
EGD: Esophagogastroduodenoscopy.
